# Lipidomic changes in the rat hippocampus following cocaine conditioning, extinction, and reinstatement of drug‐seeking

**DOI:** 10.1002/brb3.1451

**Published:** 2019-11-07

**Authors:** Sumitra Pati, Peggi Angel, Richard R. Drake, John J. Wagner, Brian S. Cummings

**Affiliations:** ^1^ Department of Pharmaceutical and Biomedical Sciences University of Georgia Athens GA USA; ^2^ Department of Cell and Molecular Pharmacology Medical University of South Carolina Charleston SC USA; ^3^ Department of Physiology and Pharmacology University of Georgia Athens GA USA; ^4^ Interdisciplinary Toxicology Program University of Georgia Athens GA USA; ^5^ Present address: Eshelman Institute for Innovation UNC Eshelman School of Pharmacy Chapel Hill NC USA

**Keywords:** addiction, hippocampus, lipidomics, lipids, mass spectrometry imaging

## Abstract

**Introduction:**

Cocaine dependence affects millions of individuals worldwide; however, there are no pharmacotherapeutic and/or diagnostic solutions. Recent evidence suggests a role for lipid signaling in the development and maintenance of addiction, highlighting the need to understand how lipid remodeling mediates neuroadaptation after cocaine exposure.

**Methods:**

This study utilized shotgun lipidomics to assess cocaine‐induced lipid remodeling in rats using a novel behavioral regimen that incorporated multiple sessions of extinction training and reinstatement testing.

**Results:**

Mass spectrometric imaging demonstrated widespread decreases in phospholipid (PL) abundance throughout the brain, and high‐spatial resolution matrix‐assisted laser desorption/ionization Fourier‐transform ion cyclotron resonance mass spectrometry indicated hippocampus‐specific PL alterations following cocaine exposure. We analyzed the expression of genes involved in hippocampal lipid metabolism and observed region‐specific regulation. In addition, we found that cocaine exposure differentially regulates mitochondrial biogenesis in the brain.

**Conclusions:**

This work presents a comprehensive lipidomic assessment of cocaine‐induced lipid remodeling in the rat brain. Further, these findings indicate a potential interplay between CNS energetics and differential lipid regulation and suggest a role for cocaine in the maintenance of energy homeostasis.

## Graphical abstract

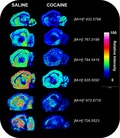

**Highlights**

- Cocaine exposure decreases phospholipid abundance throughout the brain with hippocampus‐specific alterations.
- Gene expression related to hippocampal lipid metabolism is altered region‐specifically.
- Exposure to cocaine differentially regulates mitochondrial biogenesis in the brain.

## 1 INTRODUCTION

Cocaine use disorder (CUD) is a worldwide public health concern that affects more than a million users in the United States alone (National Survey on Drug Use and Health, SAMSA, 2015). Presently, there are no FDA‐approved pharmacotherapies available for the treatment of CUD nor are there early detection strategies for its diagnosis. This highlights the need to search and identify new therapeutic targets as well as markers of exposure and dependence.

Recent studies have suggested roles for lipid signaling in the development and maintenance of addiction (Leishman, Kokesh, & Bradshaw, 2013). This supports the premise that a better understanding of lipid signaling may offer new therapeutic targets for cocaine addiction (Orio et al., 2013). Lipids are essential biomolecules that play a major role in the brain through their participation in signaling, as modulators of synaptic transmission, and are thought to play a role in the synaptic changes that are hypothesized to underlie the process of drug addiction (Hillard, 2005). Several studies have suggested that aberrant lipid metabolism occurs following exposure to drugs of abuse such as cocaine (Buydens‐Branchey & Branchey, 2003; Buydens‐Branchey, Branchey, McMakin, & Hibbeln, 2003; Zhang & Reith, 1996; Zimmer et al., 2000). A study in human patients suggested an association between cocaine dependence and increases in brain phospholipid (PL) precursors, suggesting that PLs may mediate some of the cocaine‐induced effects (Ross et al., 2002). Additionally, pathways which stimulate the release of arachidonic acid, such as activators of phospholipase A_2_, were shown to exhibit inhibitory effects on dopamine uptake, while dietary deficiency in α‐linolenic acid, the precursor of long‐chain n‐3 polyunsaturated fatty acids (PUFA), altered dopaminergic neurotransmission (Zhang & Reith, 1996; Zimmer et al., 2000). Lipid remodeling following cocaine exposure has also been linked with behaviors such as drug relapse and behavioral sensitization (Buydens‐Branchey et al., 2003; Cummings et al., 2015). Our prior work was the first to report that cocaine induces region‐specific changes in select lipid species in the rat brain and the blood using a preclinical model of drug exposure resulting in behavioral sensitization (Cummings et al., 2015).

The above studies suggest that cocaine can induce lipid ehavioural, which would contribute to cocaine's ability to induce neuroadaptation. Recent work has also revealed that lipids are also essential for mediating mitochondrial dynamics (Mårtensson, Doan, & Becker, 2017). These organelles carry critical enzymes for multiple biosynthetic processes including those essential for lipid metabolism (Hock & Kralli, 2009). Further, efficient mitochondrial function is essential for proper central nervous system performance. Interestingly, it has been suggested that drugs of abuse may directly influence the mitochondria and that mitochondrial perturbations are involved in the neurological changes associated with drug addiction (Feng et al., 2013; Sadakierska‐Chudy, Frankowska, & Filip, 2014). In a clinical study, cocaine abusers exhibited altered mitochondrial biogenesis and inhibited oxidative phosphorylation, along with energy consumption and metabolic deficits resulting in neuronal impairment (Samikkannu, Atluri, & Nair, 2016). Although the mechanisms that underlie these drug‐induced alterations are not well understood, they support the hypothesis that cocaine exposure mediates alterations to lipid metabolism and mitochondrial function.

Following our initial report (Cummings et al., 2015), other investigators (Bodzon‐Kulakowska et al., 2017; Lin et al., 2017) have recently explored the effects of drug exposure on the brain lipidome. Here, in the present study, we assess brain region‐specific lipid remodeling within a rodent model of drug exposure. We utilized lipidomics and mass spectrometric imaging to elucidate alterations in the rat lipidome along with their neuroanatomical localization following cocaine exposure. Finally, we investigated the region‐specific effects of repeated cocaine exposure on the regulation of fatty acid metabolism and mitochondrial biogenesis.

## 2 MATERIALS AND METHODS

### 2.1 Animal maintenance

Adult (12–13 weeks) male Sprague Dawley rats (Harlan) were housed in pairs in clear plastic cages and maintained on a 12‐hr light/dark cycle (0700/1900 hr) with food and water available ad libitum. Animals were allowed to acclimate to their home cages for at least 1 week and were habituated to handling (3 days) prior to testing. All studies were approved by the University of Georgia Institutional Animal Care and Use Committee and were conducted in accordance with the Guide for the Care and Use of Laboratory Animals.

### 2.2 Drug

Cocaine hydrochloride was obtained from the National Institutes of Health National Institute on Drug Abuse (RTI International) and was dissolved in 0.9% saline and filter sterilized prior to use. Animals were injected intraperitoneally with either cocaine (15 mg/kg on conditioning days, 10 mg/kg on reinstatement days) or 0.9% saline.

### 2.3 Conditioned place preference apparatus

Behavioral testing took place in 43.2‐cm × 43.2‐cm chambers with clear plastic walls and a smooth solid floor (Med Associates Inc.), as described previously (Scholpa, Briggs, Wagner, & Cummings, 2016). Briefly, each chamber was located in a sound‐attenuating box equipped with two house lights, ventilation fan, and photo beam banks for horizontal activity detection. Beam breaks were counted using Activity Monitor software (Med Associates, Inc.). A modified open field chamber with two compartments (Med Associates, Inc.) was used for conditioned place preference (CPP) testing. The chamber was divided into two compartments with a black partition containing a guillotine door. Removal of the door permitted the rat access to either compartment. For conditioning sessions, the door was closed in order to confine the animal's activity. To obtain equal preference between compartments, one compartment contained rod‐like steel bar flooring and black plastic walls while the other compartment contained a wire mesh grid floor and transparent walls.

### 2.4 Conditioned place preference training

**Figure 1:**
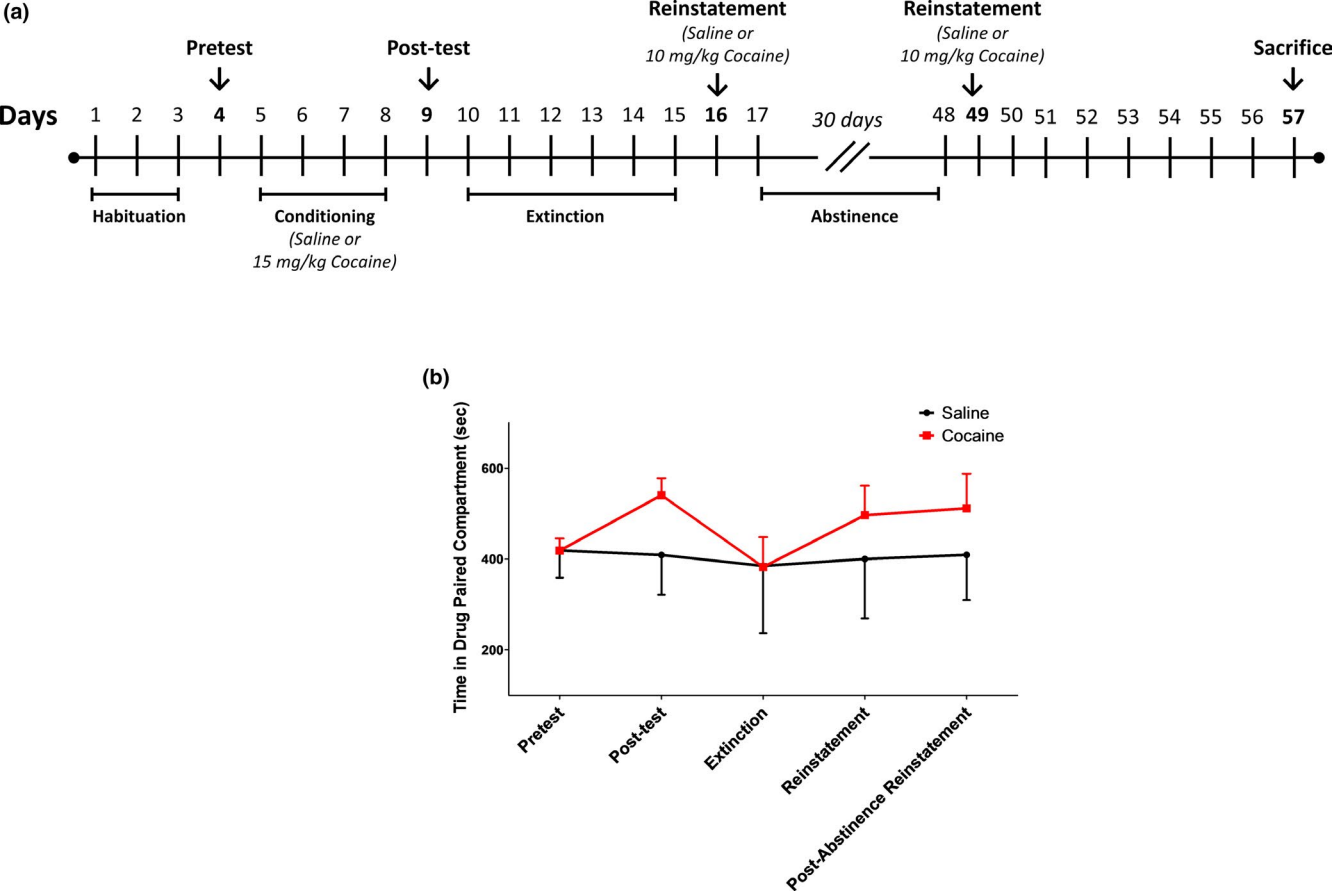
(a) Noncontingent drug exposure behavioral protocol with multiple sessions of extinction training, abstinence, and reinstatement testing. (b) Effect of cocaine on conditioned place preference. Rats were injected I.P. with either saline or cocaine under conditions shown to induce conditioned place preference (CPP). Cocaine‐induced CPP was examined in saline‐ and cocaine‐treated rats. Behavioral data are indicative of a total of eight saline‐treated (*n* = 8) and 19 cocaine‐treated (*n* = 19) rats, and data are presented as the mean ± *SEM*

The experimental design for the ehavioural protocol (Figure 1a) was modified based on that described in previous studies (Scholpa et al., 2016; Seymour & Wagner, 2008). Extinction, reinstatement, and prolonged abstinence intervals were incorporated to establish a model resembling the pattern of repeat relapse observed in humans. For the place preference assay, rats were first tested in a pretest session (day 4), during which each rat was placed in the lighter/grid floor compartment and the guillotine door was removed, allowing free access to both compartments for 15 min. The time spent in each compartment was measured to ensure that no animals had a strong bias (>65%) toward one side or another.

Conditioning sessions took place for four consecutive days (day 5–8). Rats were divided into two groups: saline‐treated (*n* = 8) and cocaine‐treated (*n* = 19). Animals were injected with either saline or cocaine (15 mg/kg, I.P.), placed in one of the two insert compartments for 15 min, and then returned to their home cage. Four hours later, animals were injected with either saline or cocaine and were confined to the opposite compartment for the second daily conditioning session. After 4 days of conditioning, the rats underwent a drug‐free place preference post‐test on experiment day 9.

Extinction and reinstatement are commonly used to model relapse, as reviewed in Aguilar, Rodriguez‐Arias, and Minarro (2009). Following the post‐test on experiment day 9, rats were allowed to move freely between compartments for 15 min. This extinction phase took place for 6 consecutive days (days 10–15) to extinguish place preference. Reinstatement was tested the day following the extinction phase (day 16). The reinstatement day was identical to the post‐test session; however, rats were injected with a cocaine prime (10 mg/kg, I.P.) immediately before being placed in the apparatus and allowed to move freely between compartments for 15 min. Following the reinstatement session on day 16, rats were left in their home cage for 30 consecutive days, with weekly handling, for a month‐long abstinence period. A repeat of the drug‐induced reinstatement test took place on experiment day 49.

### 2.5 Tissue harvesting

Region‐specific brain and liver tissues were extracted 7 days after the final exposure to cocaine, as described previously (Scholpa et al., 2016). Briefly, animals were anesthetized with halothane prior to decapitation in compliance with protocols approved by the University of Georgia Animal Care and Use guidelines. The brain was removed from the cranium, and regions of interest were extracted and flash‐frozen in liquid nitrogen. For matrix‐assisted laser desorption/ionization Fourier‐transform ion cyclotron resonance mass spectrometry (MALDI‐FTICR‐MS) studies (*n* ≥ 3 rats per group), the left hemisphere was placed midline down on a coverslip, flash‐frozen in liquid nitrogen, and kept at −80°C until cryosectioning.

### 2.6 2‐D imaging with MALDI‐FTICR MS

Fresh frozen tissue was cryosectioned into sagittal sections (10–12 µm) and thaw mounted on indium tin oxide coated slides. Tissues were sprayed with the matrix 5 mg/ml 2,5‐diaminonaphthalene in 90% acetonitrile. Matrix was applied using a TM Sprayer (HTX Imaging) with 10 passes at a 1,300 mm/min velocity and spray maintained at 60°C. Images were collected by MALDI‐FTICR (7.0 solariX Legacy; Bruker Daltonics) equipped with a Smartbeam2 laser using 200 laser shots per pixel at a 125 µm stepsize. Data were acquired in negative or positive ion mode on the same tissue, offsetting x and y steps by 75 µm. Transients of 1 Mega word were acquired in broadband mode over *m*/*z* 200–1,800 resulting in an estimated 118,000 resolving power on tissue at *m*/*z* 790 calculated at full width half max (FWHM). Data were visualized in flexImaging 4.1 build 116 (Bruker Daltonics) and analyzed for regional distribution in SCiLS Lab software version 2016b.

### 2.7 Feature selection of spectral data

For MALDI‐FTICR‐MS imaging of brain tissue, technical replicate analysis calculated values on all spectra to consider technical variability where individual spectra represent technical replicates. The maximum area under the curve (AUC) was obtained based on the receiver operating curve characteristic (ROC), and a ROC cutoff ≥0.7 was used to filter for species that discriminate between biological states. *p*‐values calculated based on the Wilcoxon Rank Sum (WRS) were classified based on a significance rating. Mean intensities from each region and area under the peak are reported per region. Values were normalized to combined total ion current and number of spectral counts, equal to number of pixels, to minimize sampling variability.

### 2.8 Real‐time quantitative PCR

RNA was isolated region‐specifically from flash‐frozen tissue using the TRIzol (Invitrogen), which was followed by the RNeasy MinElute Cleanup Kit (Qiagen) according to the manufacturer's instructions. For array analyses, RNA concentration and quality were assessed using the Bioanalyzer 2100 (Agilent Technologies) following the manufacturer's protocol and samples with RNA scores >8.0 were used for quantitative PCR (qPCR). First‐strand cDNA synthesis was performed using the RT^2^ First Strand Kit (SABiosciences) following the manufacturer's protocol.

Real‐time PCR was performed using the RT^2^ Profiler™ PCR Array: Rat Fatty Acid Metabolism Plate (SABiosciences™) according to the manufacturer's instructions, and quantitative PCR was carried out using the CFX Connect (Bio‐Rad). Treatment differences were calculated as a fold change by the ^ΔΔ^Ct method and normalized by the house‐keeping genes Actb, B2m, Hprt1, Ldha, and Rplp1. Unsupervised hierarchical clustering of the dataset was performed by clustergram analysis to display a heat map with dendrograms indicating co‐regulated genes across groups and samples with using the RT^2^ profiler RT‐PCR array data analysis software, version 3.5.

Array results were validated with qPCR on altered fatty acid metabolism targets and mitochondrial biogenesis genes. Total RNA (100 ng) was used for cDNA synthesis using the iScript cDNA Synthesis Kit (Bio‐Rad). cDNA (100 ng) with a 260:280 >1.8 was used for quantitative PCR using iTaq Universal SYBR Green Supermix (Bio‐Rad) with specific primers (Table S1).

### 2.9 Statistical analyses and data sharing

All statistical analyses were compiled using GraphPad Prism for windows version 5.04 (GraphPad Software). For all analysis, the experimental unit was individual subjects and samples obtained from a minimum of 4–5 subjects/group were assessed. For all analyses, significance was set at *p* ≤ .05 where data are expressed as mean ± *SEM* based on *t* test for pairwise analysis and/or ANOVA analysis (two‐factor repeated‐measures with Bonferroni post hoc test). The data that support the findings of this study are available from the corresponding author upon reasonable request.

## 3 RESULTS

### 3.1 Conditioned place preference model

We modified a behavioral model based on that described in previous studies (Scholpa et al., 2016; Seymour & Wagner, 2008) that incorporated extinction, reinstatement, and prolonged abstinence in order to model a pattern of relapse representative of what might be observed in humans that use cocaine (Figure 1a). Cocaine‐treated rats exhibited place preference following conditioning as indicated by more time spent in the designated drug‐paired compartment during the CPP post‐test than during the pretest (Figure 1b). Although cocaine‐treated rats demonstrated varied CPP response, cocaine‐treated rats spent more time in the drug‐paired compartment during reinstatement testing as compared to the outcomes of extinction testing.

### 3.2 Localization of cocaine‐induced brain lipid alterations with mass spectrometric imaging

**Figure 2:**
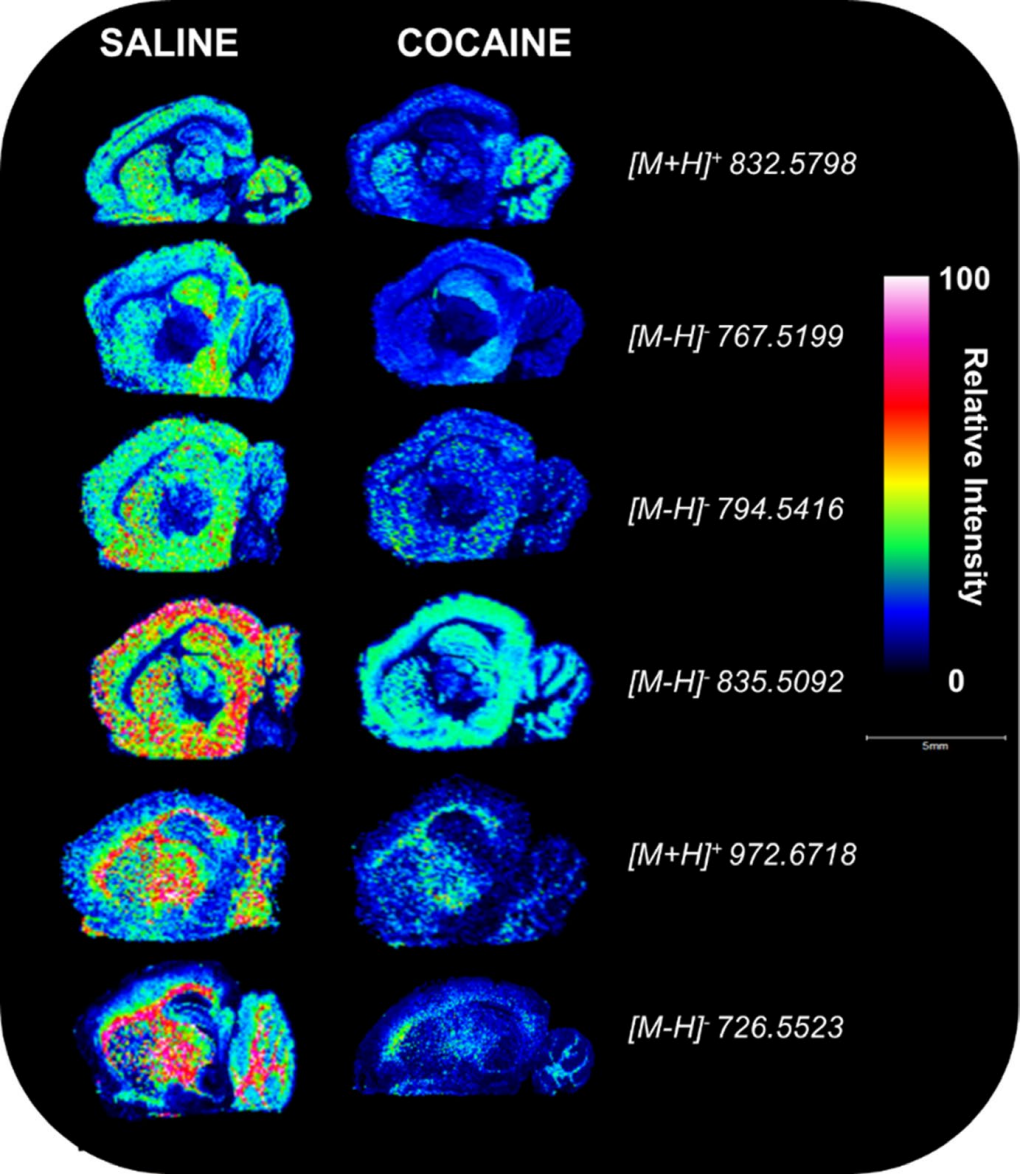
Differential abundance and distribution of phospholipid species throughout rat brain following cocaine exposure with matrix‐assisted laser desorption/ionization Fourier‐transform ion cyclotron resonance mass spectrometry (MALDI‐FTICR‐MS) analysis. Rats were injected I.P. with either saline or cocaine as described previously. The left‐brain hemisphere was extracted 7 days after the final treatment (day 57), and sagittal sections were analyzed by MALDI‐FTICR‐MS analysis. Sections were assessed from saline‐treated (left) and cocaine‐treated (right) rats. Representative images are shown from each group to illustrate trends observed in tissue sections from at least three different rats. Color scale represents the relative intensity normalized to total ion count. Scale bar: 5 mm

Low spatial resolution MALDI‐FTICR‐MS imaging was performed on entire, sagittal brain sections from saline‐ and cocaine‐treated rats to understand relative lipid expression and distribution. This analysis demonstrated decreases in lipid species in the *m*/*z* range from 700 to 1,000 in cocaine‐treated rats as compared to saline controls (Figure 2). The representative two‐dimensional images of spectral data indicate decreases in the relative abundance of lipid species throughout structures such as the cerebellum, hippocampus, striatum, corpus callosum, frontal cortex, and the thalamus (Figure 2).

### 3.3 Hippocampus‐specific PL alterations following cocaine exposure

**Figure 3:**
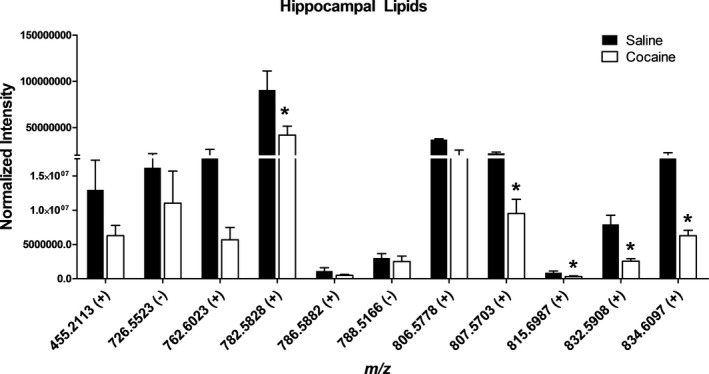
Normalized abundance of hippocampus‐specific lipid features altered following cocaine exposure. Rats were injected I.P. with either saline or cocaine as described previously. The left‐brain hemisphere was extracted 7 days after the final treatment (day 57), and sagittal sections were analyzed by matrix‐assisted laser desorption/ionization Fourier‐transform ion cyclotron resonance mass spectrometry (MALDI‐FTICR‐MS) analysis. Mean intensities from each region and area under the peak were obtained from high‐spatial resolution MALDI‐FTICR‐MS analysis of the hippocampus and normalized to combined total ion current and number of spectral counts, equal to number of pixels. Features were filtered based on an ROC cutoff ≥0.7 and/or *p*‐value ≤0.05 (*Significant difference based on both ROC and *p*‐value as compared to saline‐treated rats). The data are presented as the mean ± *SEM* of tissue sections from at least three different rats

As we have previously observed lipid changes in the hippocampus of cocaine‐exposed rats (Cummings et al., 2015), we performed further assessment of that brain region. High‐spatial resolution MALDI‐FTICR‐MS analyses of the hippocampal region showed decreases in lipid abundances for species in the *m*/*z* range from 700 to 900 along with a lower molecular weight lipid at *m*/*z* 455.2113 following cocaine exposure (Figure 3). Statistical feature identification of hippocampal lipids was based on ROC filtering and *p*‐value comparisons between cocaine‐ and saline‐treated rats. Five lipids significantly decreased in cocaine‐treated rats: *m*/*z* 782.5828, *m*/*z* 807.5703, *m*/*z* 815.6987, *m*/*z* 832.5908, and *m*/*z* 834.6097 (Figure 3).

### 3.4 Effect of cocaine exposure on fatty acid metabolism regulation

**Figure 4:**
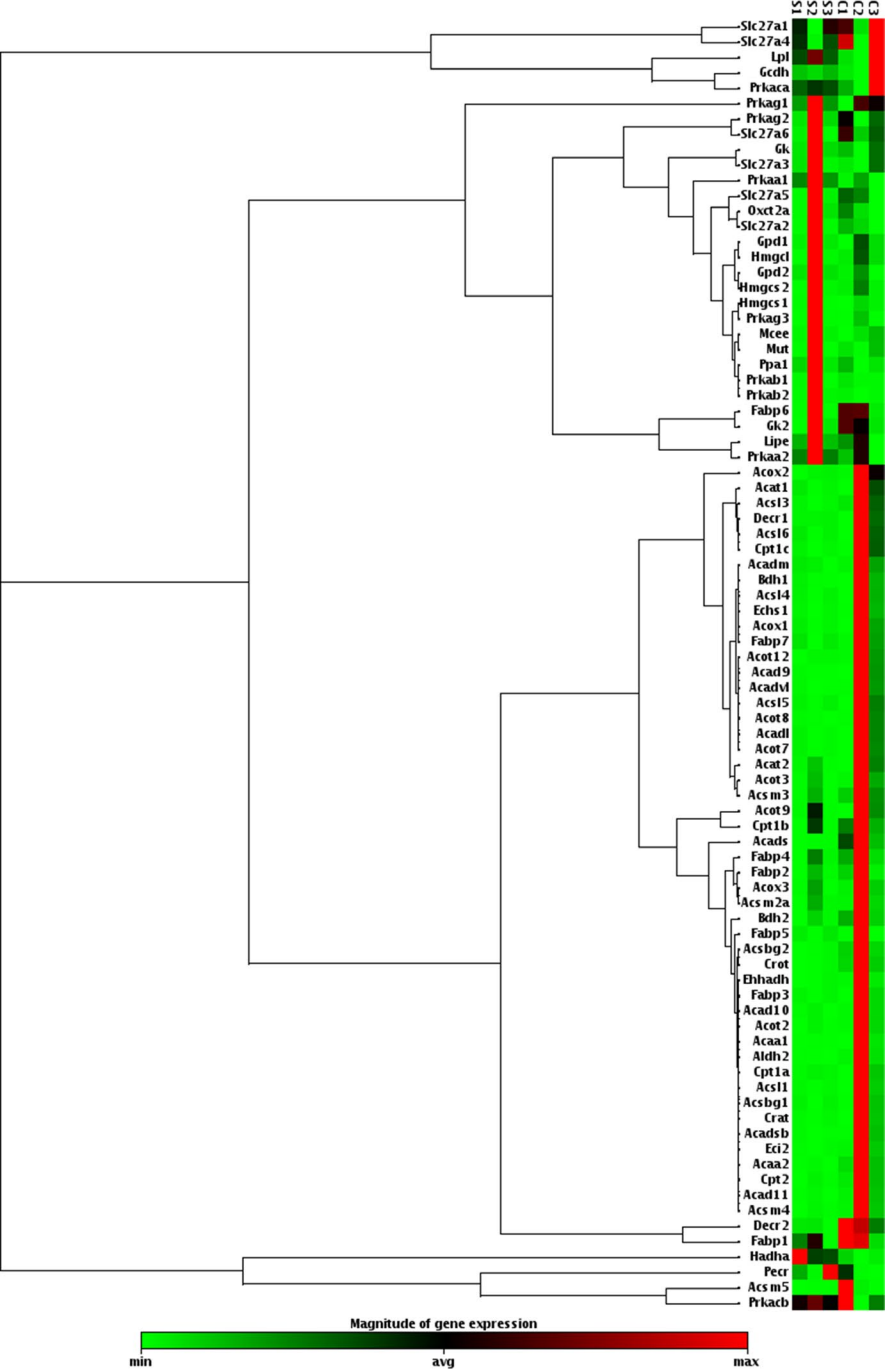
Clustergram analysis and heatmap graph of gene expression data. Hippocampal expression of 84 fatty acid metabolism‐related genes is represented as a heatmap graph with genes clustered according to their expression patterns. Rats were injected I.P. with either saline or cocaine as described previously. Hippocampal tissue was extracted from the right‐brain hemisphere 7 days after the final treatment (day 57) and assessed for mRNA expression of an 84‐set list of genes related to fatty acid metabolism using qPCR. The data are indicative of tissue from at least three different rats per group

**Figure 5:**
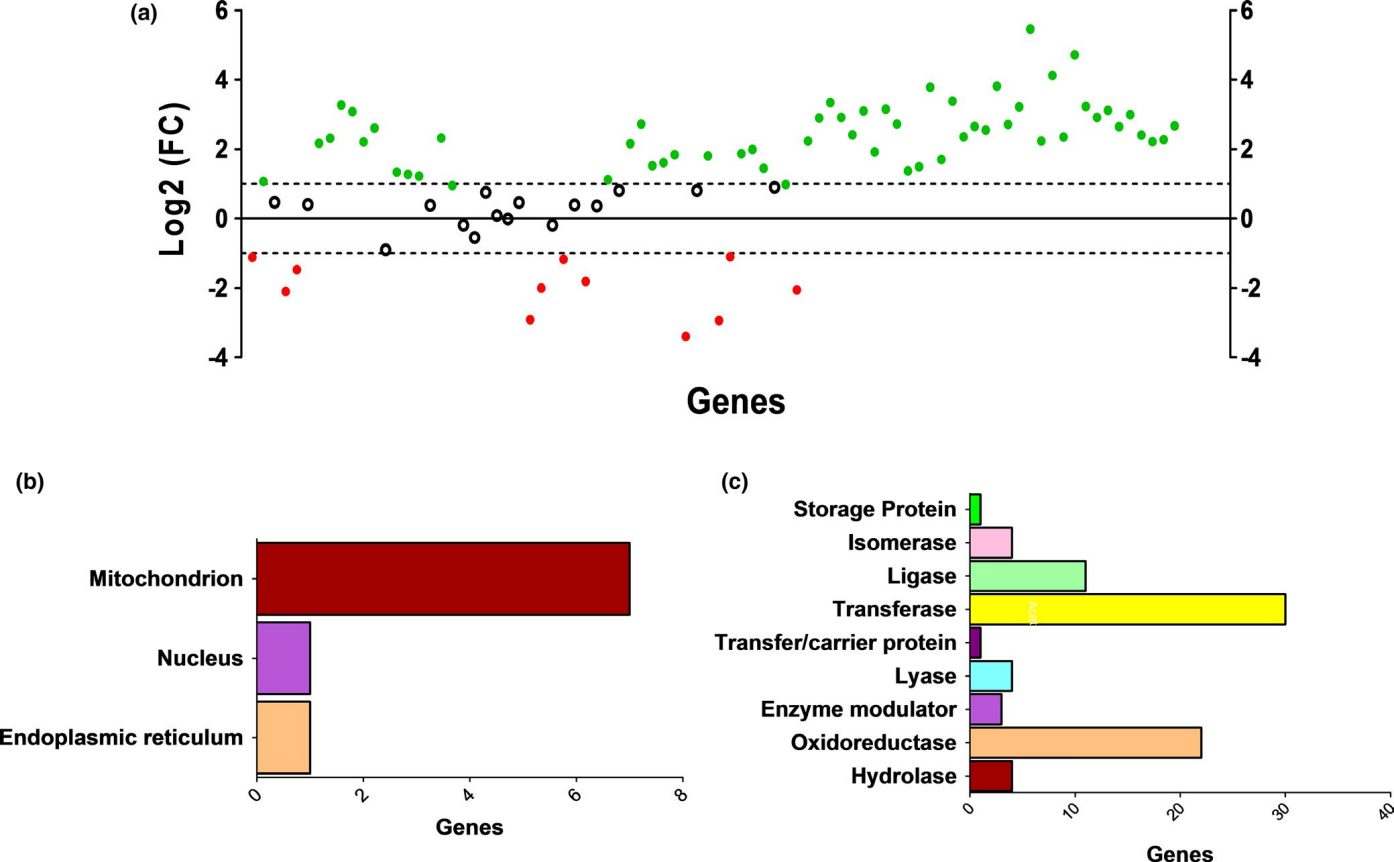
Volcano plot analysis and pathway analysis of fatty acid metabolism‐related genes in the rat hippocampus following cocaine exposure. (a) Volcano plot analysis indicated that cocaine treatment upregulated 68 genes and downregulated 16 genes related to fatty acid metabolism and biosynthesis. The PANTHER database was used to analyze upregulated genes in the rat hippocampus to identify (b) protein class and (c) cellular compartmentalization

Although our data indicate that cocaine exposure decreases PLs abundance in the brain, we did not observe increases in fatty acid (FA) species that correspond to PL remodeling. This phenomenon suggested a potential role for perturbed regulation of genes involved in fatty acid metabolism. We analyzed the gene expression of hippocampal fatty acid metabolism using a gene expression array of tissue comparing cocaine‐ and saline‐treated rats. Based on the expression of 84 fatty acid metabolism genes (Figure 4), a pattern of upregulation was observed in 68 genes in the hippocampal tissue of cocaine‐treated rats with respect to saline‐treated controls (Figure 5a, Table S2). Sixteen genes were downregulated in cocaine‐treated rats, many of which with regulatory functions for protein kinases and fatty acid transporters, for example, members of the solute carrier family (Figure 5, Table S2). Protein class and cellular compartmentalization were also determined for the upregulated genes via biological function analyses (http://www.pantherdb.org), indicating that many of the upregulated genes encode for transferase proteins, specifically acetyltransferases (Figure 5b), several of which are mitochondrial (Figure 5c).

**Figure 6:**
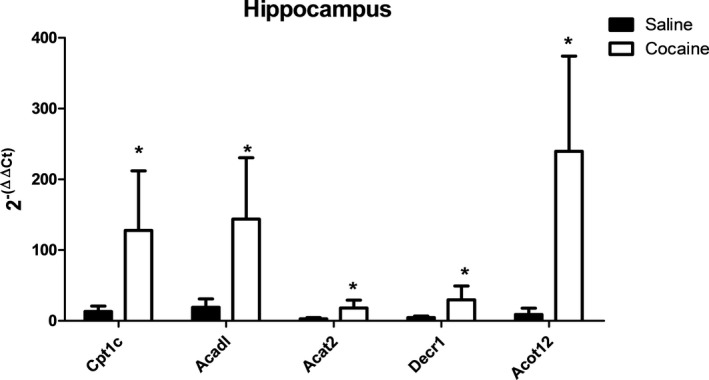
Effect of cocaine exposure on genes related to fatty acid metabolism in the rat hippocampus. The indicated tissues were extracted 7 days after the final treatment (day 57) and assessed for mRNA expression of carnitine palmitoyltransferase 1C (Cpt1c), acyl‐CoA dehydrogenase (Acadl), acetyl‐CoA acetyltransferase (Acat2), 2,4‐Dienoyl‐CoA reductase (Decr1), and acyl‐coenzyme A thioesterase 12 (Acot12) using qPCR. The data are indicative of tissue from at least three different rats per group and are expressed as 2^−(ΔΔCt)^ ± *SEM* ΔCt. *Significant difference (*p* < .05) as compared to saline‐treated controls

Significantly altered genes identified from the hippocampal array analyses were individually validated with qPCR (Figure 6). These genes consisted of (a) carnitine palmitoyltransferase 1C (Cpt1c), (b) acyl‐CoA dehydrogenase (Acadl), (c) acetyl‐CoA acetyltransferase (Acat2), (d) 2,4‐Dienoyl‐CoA reductase (Decr1), and (e) acyl‐coenzyme A thioesterase 12 (Acot12). qPCR analyses of these genes (Figure 6) confirmed findings from the array analysis, while analysis of gene expression in the cerebellum (Figure S1a) demonstrated a significant ~twofold downregulation of the Acat2 gene in cocaine‐treated rats. The effects of cocaine exposure were also assessed in peripheral liver tissue (Figure S1b) of the 5 targets identified in the brain. These data showed a trend of numerical decreases, with significant downregulation of the Acadl gene following cocaine exposure, and undetected expression of Cpt1c (Figure S1b).

### 3.5 Regulation of mitochondrial biogenesis in the brain and liver following cocaine exposure

**Figure 7:**
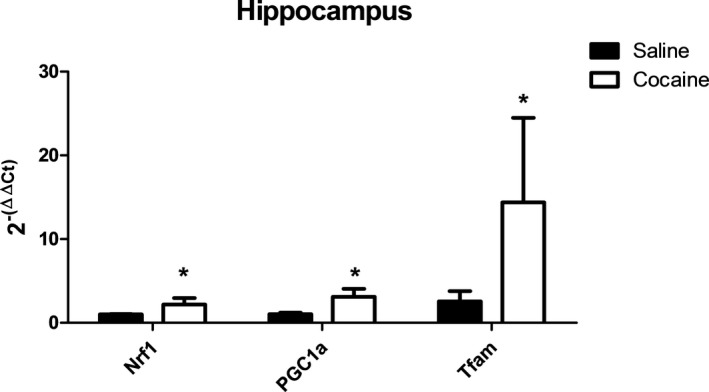
Effect of cocaine exposure on mitochondrial biogenesis genes in the rat hippocampus. The indicated tissues were extracted 7 days after the final treatment (day 57) and assessed for mRNA expression of nuclear respiratory factor 1 (Nrf1), peroxisome proliferator‐activated receptor gamma coactivator 1‐alpha (PGC‐1α), and transcription factor A, mitochondrial (Tfam) using qPCR. The data are indicative of tissue from at least three different rats per group and are expressed as 2^−(ΔΔC^ *^t^* ^)^ ± *SEM* ΔCt. *Significant difference (*p* < .05) as compared to saline‐treated controls

We assessed the effect of repeated cocaine exposure on mitochondrial biogenesis and the growth and division of pre‐existing mitochondria to accommodate increases in ATP production as a response to greater energy expenditure (Jornayvaz & Shulman, 2010). Specifically, we assessed markers of mitochondrial biogenesis, a highly regulated process mediated by peroxisome proliferation‐activated receptor coactivator 1 α (PGC‐1α), PGC‐1α‐dependent nuclear respiratory factors (NRFs), and mitochondria transcription factor A (Tfam). Gene expression of these markers was all significantly upregulated in the hippocampus in cocaine‐treated rats (Figure 7). This was not paralleled in the cerebellum, which indicated a trend of numerical decreases in tissue from cocaine‐treated rats (Figure S2a). Remarkably, these genes were all significantly downregulated in the liver following cocaine exposure with the most pronounced decrease in PGC‐1α (Figure S2b).

## 4 DISCUSSION

Cocaine is a powerfully addictive stimulant drug whose abuse can lead to dependence, a chronic relapsing disease caused by changes in the brain, and is lacking in pharmacotherapeutic and diagnostic solutions. There is increasing evidence that implicates a role for lipids as related to drugs of abuse, highlighting the need for lipidomic research that may reveal new targets for drug development and/or biomarker detection (Buydens‐Branchey & Branchey, 2003; Buydens‐Branchey et al., 2003; Zhang & Reith, 1996; Zimmer et al., 2000). Such research is also essential for expanding our current understanding of how lipids are involved in signal transduction processes accompanying the neurobiology of addiction. Recent advances in the development of mass spectrometric tools to characterize molecular lipids have made it possible to unravel the complexity of the lipidome along with the perturbations in lipid metabolism which occur in numerous diseases such as obesity, diabetes, and Alzheimer's disease (Steinberg, 2005; Watson, 2006; Wenk, 2005).

The behavioral protocol used in this study was intended to model the pattern of repeated relapse often observed in humans by incorporating intervals of extinction, reinstatement, and prolonged abstinence, which were assessed using CPP. Using this model, cocaine‐treated rats exhibited place preference within each behavioral session, although rats displayed differential CPP response. While variability in CPP is a common behavioral phenomenon in drugs of abuse studies conducted in rodents, these outcomes may resemble the variation in drug response observed in humans. However, our previous work demonstrating correlations between lipid relative abundance and differential locomotor sensitization suggest that in some instances, behavioral variability is reflected downstream in peripheral biological processes (Cummings et al., 2015).

A striking overlap between both our current and previous studies (Cummings et al., 2015) was that the majority of lipid alterations occurred in the hippocampus and cerebellum, with relatively few changes in the striatum. However, our previous study used shotgun electrospray ionization‐MS to assess whole‐scale changes in the isolated regions. The use of mass spectrometric imaging (MSI) confirms these data and demonstrates widespread lipid alterations throughout rat brain substructures. Specific changes in the hippocampus were not surprising given the involvement of this region in learning and memory, which is associated with drug‐seeking behavior through the conditioned cues or environmental context that accompany drug experiences (Fuchs et al., 2005; Sun & Rebec, 2003). The PL alterations detected in the hippocampus suggest a role for lipids in the retention of the memories of addiction, possibly due to membrane remodeling. Lipidomic changes in the cerebellum were notable since this region was previously presumed to lack relevance to the mechanisms underlying addictive behavior. However, the current study, along with our previously mentioned work, is among the first to suggest a role for the cerebellum in drug reward and reinstatement of drug‐seeking (Koob & Volkow, 2010; Moreno‐Rius & Miquel, 2017; Moulton, Elman, Becerra, Goldstein, & Borsook, 2014; Wagner, Kim, Savall, Schnitzer, & Luo, 2017). Other studies have recently reported consistent cerebellar activation in response to drug cues via neuroimaging and bidirectional connections with regions mediating drug reward and involvement in nonmotor function (Moreno‐Rius & Miquel, 2017; Moulton et al., 2014; Wagner et al., 2017).

As mentioned above, MSI clearly demonstrated cocaine‐induced lipid remodeling throughout rat brain substructures. The finding that cocaine exposure reduces PL levels throughout the brain and hippocampus was interesting given the findings of Ross et al. (2002) who showed decreased activity of key PL‐metabolizing enzymes, calcium‐stimulated phospholipase A_2_, and phosphatidylcholine cytidylyltransferase, in dopaminergic brain regions of human cocaine users.

Although absolute quantitation was not performed for PL features, the data are likely an accurate indication of lipid changes following cocaine exposure. Lipid concentrations obtained by MALDI‐MSI signals recently showed successful comparisons to those assessed by LC‐MS/MS in brain tissue (Hankin & Murphy, 2010). A few studies have used MSI to analyze cocaine drug and metabolites (Pirman, Reich, Kiss, Heeren, & Yost, 2012; Wang, Jackson, McEuen, & Woods, 2005); however, the current study is one of the first to report the persisting effects of cocaine on differential lipid abundance with MSI. A recent study by Bodzon‐Kulakowska et al. (2017) demonstrated the influence of cocaine, morphine, and amphetamine on select PL species throughout limbic structures using desorption electrospray MSI. Comparison of this study to ours demonstrated a few of the same PLs alterations (*m*/*z* 834.5, *m*/*z* 786.5, *m*/*z* 806.6). These similarities occurred despite differences between the behavioral regimens used as well as differences in the dose and frequency of cocaine administration (Bodzon‐Kulakowska et al., 2017). Another recent study showed the profound effect of cocaine on the brain lipidome in mice using behavioral models of CPP and locomotor sensitization (Lin et al., 2017). Lin et al. (2017) demonstrated significant decreases in ceramide and lysophospholipids accompanied by increases in PLs and polyunsaturated fatty acid (PUFA)‐containing PLs in the nucleus accumbens.

Although our data demonstrate the finding that cocaine persistently induces decreases in PLs throughout various brain structures, corresponding increases in FA species were not observed. We have previously demonstrated that oxidant‐induced damage in neurons results in increased FA release that accompanied by PL remodeling, as indicated by increased levels of PLs with PUFAs (Peterson, Stovall, Monian, Franklin, & Cummings, 2008). The absence of such remodeling in the current study raises a question regarding how the synthesis and metabolism of these fatty acids are being affected by cocaine. The mitochondria are a key site for fatty acid metabolism, and recent work suggests that energy deficits play a regulatory role in drug addiction and in the development of neurodegenerative diseases (Cunnane et al., 2011; Thanos, Michaelides, Benveniste, Wang, & Volkow, 2008). Studies have also shown that cocaine impacts glucose metabolism with subsequent effects on the brain (Renthal & Nestler, 2008). These findings, coupled with the active involvement of the mitochondria in the regulation of synaptic plasticity, led us to investigate the role of cocaine on the differential regulation of mitochondrial biogenesis and fatty acid metabolism. Our observation that cocaine exposure upregulates hippocampal FA metabolism supports the hypothesis that cocaine alters the maintenance of energy homeostasis. Moreover, the differences between hippocampal and cerebellar expression of genes involved in FA metabolism indicate that such regulation occurs region‐specifically. The trend of downregulation observed for these FA gene targets in the liver was interesting, although further characterization of the effect of cocaine on peripheral FA metabolism is beyond the scope of the current work.

Phospholipid synthesis involves several enzymes, many of which are interlinked. While a direct correspondence between the reduction in the PLs identified in this study and the increase in enzymes involved in fatty acid metabolism cannot be made, the reduction in the PLs identified may be countered by a change in fatty acid metabolism. This aligns with the increase in mRNA levels Cpt1c, Acad1, and Act2, which would be needed to synthesize fatty acids and to add substituents, enabling these fatty acids to be incorporated into PL precursors. These data show that an inverse relationship appears to exist between the reduction in PLs induced by cocaine and an increase in enzymes responsible for fatty acid metabolism.

Recent studies suggest that mitochondrial energetics are altered during neuronal injury and protective mechanisms can be initiated by increasing mitochondrial biogenesis (Chen et al., 2011). Little attention has been given to understanding the effect of cocaine on energy expenditure via mitochondrial biogenesis. The finding that cocaine exposure upregulates markers of mitochondrial biogenesis in the hippocampus is interesting, but in line with changes in the expression of mitochondrial fatty acid regulatory genes. Further, these changes appear to be region‐specific as cerebellar and liver biogenesis was downregulated, with striking decreases in the liver. The hippocampal findings are in agreement with Sadakierska‐Chudy et al. (2017) who reported significant elevation in mtDNA copy number and concomitant increased expression of mitochondrial genes including Tfam in the hippocampus and prefrontal cortex of rats following cocaine self‐administration. However, such responses may vary based on behavior and drug exposure and may not extend to other classes of drugs of abuse. For example, Feng et al. (2013) reported an association between opioid addiction with mtDNA copy number reduction and neurostructural remodeling in the hippocampus. Further, the influence of cocaine exposure on the mitochondria has been shown to differentially affect ROS production, superoxide dismutase activity, and mitochondrial gene transcription at a variety of doses, ranging from acute to chronic, and has been reviewed in detail (Sadakierska‐Chudy et al., 2014).

In summary, the current work provides a comprehensive lipidomic assessment of cocaine‐induced alterations in the rat brain. We report hippocampus‐specific changes in the rat brain. These data suggest that behaviors resulting from cocaine conditioning, for example, sensitization, extinction, and reinstatement, are involved in the induction of widespread lipid alterations. Finally, we also show region‐specific regulation of genes involved in fatty acid metabolism and mitochondrial biogenesis in the brain and liver, demonstrating a potential interplay between CNS energetics and differential lipid regulation. Our findings link cocaine exposure to the induction of lipidomic changes in both the CNS and periphery and suggest a role for cocaine in the maintenance of energy homeostasis, providing novel insight into the link between lipids and drugs of abuse.

## CONFLICT OF INTEREST

None declared.

## Data Availability

The data that support the findings of this study are available from the corresponding author upon reasonable request.
