# Supplementary material for: Lipidomic changes in the rat hippocampus following cocaine conditioning, extinction, and reinstatement of drug‐seeking
Source: Brain Behav. 2019 Nov 7;9(12):e01451. doi: 10.1002/brb3.1451 (PMC6908860; doi:10.1002/brb3.1451)

Lipidomic Changes in the Rat Hippocampus Following Cocaine Conditioning, Extinction, and Reinstatement of Drug-seeking

Sumitra Pati[1,], Peggi Angel[2], Richard R. Drake[2], John J. Wagner[3,4], Brian S. Cummings[1,4, ¥]*

[1]Dept. of Pharmaceutical and Biomedical Sciences, University of Georgia, Athens, GA

[*]*Current Affiliation: Eshelman Institute for Innovation, UNC Eshelman School of Pharmacy, Chapel Hill, NC*

[2]Dept. of Cell and Molecular Pharmacology, Medical University of South Carolina, Charleston, SC

[3]Dept. of Physiology and Pharmacology, University of Georgia, Athens, GA

[4]Interdisciplinary Toxicology Program, University of Georgia, Athens, GA

[¥]Corresponding Author: Brian S. Cummings
336 College of Pharmacy
University of Georgia
Athens, GA 30602
Phone: 706-542-3792
Fax: 706-542-5358
E-mail: bsc@rx.uga.edu

Supplementary Table 1. Primer sequences for fatty acid metabolism and mitochondrial biogenesis genes.

**Gene Name**		**Primer Sequence**
**Nrf1**	Sense	5’-CCT CTG GGC TGT TGT GAA TTA
	Anti-sense	3’-GTC CGA GTC ATC GTA AGA AGT G
**Tfam**	Sense	5’-TGA AGT TCT TAC ACT GAT GGC
	Anti-sense	3’-CCA CGT CAT CTA GTA AAG CC
**PGC-1α**	Sense	5’-AGG CTC AAG AGG GAC GAA TA
	Anti-sense	3’-CAC AGG TGT AAC GGT AGG TAA TG
**Acadl**	Sense	5’-TGA TTC CCT ACC ACG AAG AAT G
	Anti-sense	3’-GAG AAT CCA ATC ACT CCC AGA C
**Acat2**	Sense	5’-GGA CCG ATT CCA GCC ATA AA
	Anti-sense	3’-GGT GTG TAA CAA GGT CAC TAG AA
**Decr1**	Sense	5’-GTG ACG TTC GAG ATC CTG ATA TG
	Anti-sense	3’-ATA GGC CCT GGC TGA ATT ATG
**Acot12**	Sense	5’-CCA CCA CCT TGG AGA AGA TAA A
	Anti-sense	3’-CAC CGA GCA GGT GAT GTA AT
**Cpt1c**	Sense	5’-GAC TGG TGG GAA GAG TTT GT
	Anti-sense	3’-GAC GAT GAG GGT GAA GGA TTT
**β-actin**	Sense	5’-CAC CCG CGA GTA CAA CCT T
	Anti-sense	3’-CCC ATA CCC ACC ATC ACA CC

Supplemental Table 2. Fold change for expression analysis of rat fatty acid metabolism genes. Fold change (FC) analysis was performed based on the FC of $2^{-(\Delta\Delta Ct)}$ values comparing cocaine- and saline-treated rats. The data are indicative of tissue from at least 3 different rats per group.

Symbol	Description	Fold Change
Acaa1	Acetyl-Coenzyme A acyltransferase 1A	5.30
Acaa2	Acetyl-Coenzyme A acyltransferase 2	4.63
Acad10	Acyl-Coenzyme A dehydrogenase family, member 10	4.70
Acad11	Acyl-Coenzyme A dehydrogenase family, member 11	7.43
Acad9	Acyl-Coenzyme A dehydrogenase family, member 9	10.10
Acadl	Acyl-Coenzyme A dehydrogenase, long-chain	7.53
Acadm	Acyl-Coenzyme A dehydrogenase, C-4 to C-12 straight chain	5.31
Acads	Acyl-Coenzyme A dehydrogenase, C-2 to C-3 short chain	8.57
Acadsb	Acyl-Coenzyme A dehydrogenase, short/branched chain	3.79
Acadvl	Acyl-Coenzyme A dehydrogenase, very long chain	8.87
Acat1	Acetyl-coenzyme A acetyltransferase 1	4.84
Acat2	Acetyl-Coenzyme A acetyltransferase 3	6.37
Acot12	Acyl-CoA thioesterase 12	26.27
Acot2	Acyl-CoA thioesterase 2	9.37
Acot3	Acyl-CoA thioesterase 3	7.52
Acot7	Acyl-CoA thioesterase 7	8.67
Acot8	Acyl-CoA thioesterase 8	6.25
Acot9	Acyl-CoA thioesterase 9	7.94
Acox1	Acyl-Coenzyme A oxidase 1, palmitoyl	4.70
Acox2	Acyl-Coenzyme A oxidase 2, branched chain	17.41
Acox3	Acyl-Coenzyme A oxidase 3, pristanoyl	5.09
Acsbg1	Acyl-CoA synthetase bubblegum family member 1	2.82
Acsbg2	Acyl-CoA synthetase bubblegum family member 2	13.78
Acsl1	Acyl-CoA synthetase long-chain family member 1	3.26
Acsl3	Acyl-CoA synthetase long-chain family member 3	10.42
Acsl4	Acyl-CoA synthetase long-chain family member 4	5.11
Acsl5	Acyl-CoA synthetase long-chain family member 5	6.30
Acsl6	Acyl-CoA synthetase long-chain family member 6	5.85

Acsm2a	Acyl-CoA synthetase medium-chain family member 2	14.05
Acsm3	Acyl-CoA synthetase medium-chain family member 3	6.54
Acsm4	Acyl-CoA synthetase medium-chain family member 4	9.30
Acsm5	Acyl-CoA synthetase medium-chain family member 5	43.92
Aldh2	Aldehyde dehydrogenase 2 family (mitochondrial)	4.46
Bdh1	3-hydroxybutyrate dehydrogenase, type 1	3.65
Bdh2	3-hydroxybutyrate dehydrogenase, type 2	3.97
Cpt1a	Carnitine palmitoyltransferase 1a, liver	4.49
Cpt1b	Carnitine palmitoyltransferase 1b, muscle	4.96
Cpt1c	Carnitine palmitoyltransferase 1c	9.64
Cpt2	Carnitine palmitoyltransferase 2	8.43
Crat	Carnitine acetyltransferase	4.63
Crot	Carnitine O-octanoyltransferase	6.10
Decr1	2,4-dienoyl CoA reductase 1, mitochondrial	6.59
Decr2	2,4-dienoyl CoA reductase 2, peroxisomal	2.88
Echs1	Enoyl Coenzyme A hydratase, short chain, 1, mitochondrial	3.05
Eci2	Enoyl-Coenzyme A delta isomerase 2	3.57
Ehhadh	Enoyl-Coenzyme A, hydratase/3-hydroxyacyl Coenzyme A dehydrogenase	6.60
Fabp1	Fatty acid binding protein 1, liver	-1.87
Fabp2	Fatty acid binding protein 2, intestinal	2.52
Fabp3	Fatty acid binding protein 3, muscle and heart	2.42
Fabp4	Fatty acid binding protein 4, adipocyte	2.33
Fabp5	Fatty acid binding protein 5, epidermal	1.30
Fabp6	Fatty acid binding protein 6, ileal	4.98
Fabp7	Fatty acid binding protein 7, brain	1.94
Gcdh	Glutaryl-Coenzyme A dehydrogenase	2.59
Gk	Glycerol kinase	-2.17
Gk2	Glycerol kinase 2	2.10
Gpd1	Glycerol-3-phosphate dehydrogenase 1 (soluble)	1.38
Gpd2	Glycerol-3-phosphate dehydrogenase 2, mitochondrial	-4.28
Hadha	Hydroxyacyl-Coenzyme A dehydrogenase/3-ketoacyl-Coenzyme A thiolase/enoyl-Coenzyme A hydratase (trifunctional protein), alpha subunit	-10.52
Hmgcl	3-hydroxymethyl-3-methylglutaryl-Coenzyme A lyase	2.73
Hmgcs1	3-hydroxy-3-methylglutaryl-Coenzyme A synthase 1 (soluble)	1.86

Hmgcs2	3-hydroxy-3-methylglutaryl-Coenzyme A synthase 2 (mitochondrial)	1.97
Lipe	Lipase, hormone sensitive	-2.77
Lpl	Lipoprotein lipase	1.32
Mcee	Methylmalonyl CoA epimerase	1.75
Mut	Methylmalonyl-Coenzyme A mutase	3.49
Oxct2a	3-oxoacid CoA transferase 2A	-4.15
Pecr	Peroxisomal trans-2-enoyl-CoA reductase	-7.66
Ppa1	Pyrophosphatase (inorganic) 1	-2.14
Prkaa1	Protein kinase, AMP-activated, alpha 1 catalytic subunit	-7.54
Prkaa2	Protein kinase, AMP-activated, alpha 2 catalytic subunit	-4.01
Prkab1	Protein kinase, AMP-activated, beta 1 non-catalytic subunit	-1.14
Prkab2	Protein kinase, AMP-activated, beta 2 non-catalytic subunit	-2.26
Prkaca	Protein kinase, cAMP-dependent, catalytic, alpha	1.31
Prkacb	Protein kinase, cAMP dependent, catalytic, beta	-3.51
Prkag1	Protein kinase, AMP-activated, gamma 1 non-catalytic subunit	1.28
Prkag2	Protein kinase, AMP-activated, gamma 2 non-catalytic subunit	2.17
Prkag3	Protein kinase, AMP-activated, gamma 3 non-catalytic subunit	1.75
Slc27a1	Solute carrier family 27 (fatty acid transporter), member 1	-1.14
Slc27a2	Solute carrier family 27 (fatty acid transporter), member 2	-1.46
Slc27a3	Solute carrier family 27 (fatty acid transporter), member 3	1.69
Slc27a4	Solute carrier family 27 (fatty acid transporter), member 4	1.06
Slc27a5	Solute carrier family 27 (fatty acid transporter), member 5	-1.01
Slc27a6	Solute carrier family 27 (fatty acid transporter), member 6	1.38

Supplemental Figure 1. Effect of cocaine exposure on genes related to fatty acid metabolism in the rat **A.** cerebellum and **B.** liver. The indicated tissues were extracted seven days after the final treatment (day 57) and assessed for mRNA expression of Carnitine Palmitoyltransferase 1C (Cpt1c), Acyl-CoA Dehydrogenase (Acadl), Acetyl-CoA Acetyltransferase (Acat2), 2,4-Dienoyl-CoA Reductase (Decr1), and Acyl-coenzyme A Thioesterase 12 (Acot12) using qPCR. The data are indicative of tissue from at least 3 different rats per group and are expressed as $2^{-(\Delta\Delta Ct)}$ ± SEM ΔCt. *Indicates a significant difference ($p < 0.05$) as compared to saline-treated controls

A. Cerebellum

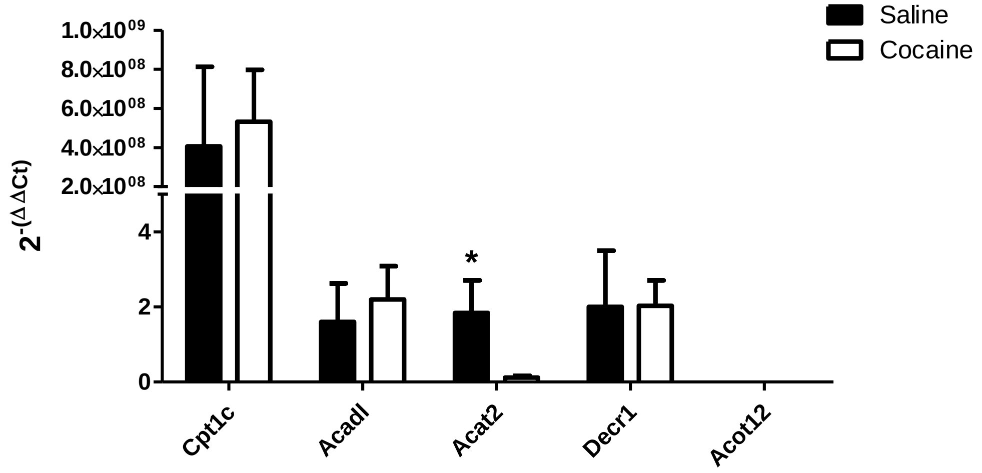

B. Liver

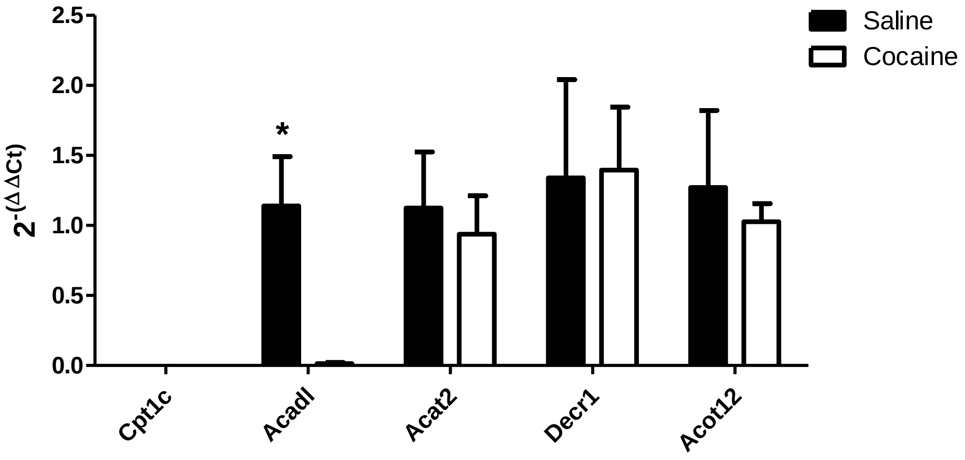

Supplemental Figure 2. Effect of cocaine exposure on mitochondrial biogenesis genes in the rat **A.** cerebellum and **B.** liver. The indicated tissues were extracted seven days after the final treatment (day 57) and assessed for mRNA expression of Nuclear respiratory factor 1 (Nrf1), Peroxisome proliferator-activated receptor gamma coactivator 1-alpha (PGC-1α), and transcription factor A, mitochondrial (Tfam) using qPCR. The data are indicative of tissue from at least 3 different rats per group and are expressed as $2^{-(\Delta\Delta Ct)}$ ± SEM ΔCt. *Indicates a significant difference ($p < 0.05$) as compared to saline-treated controls.

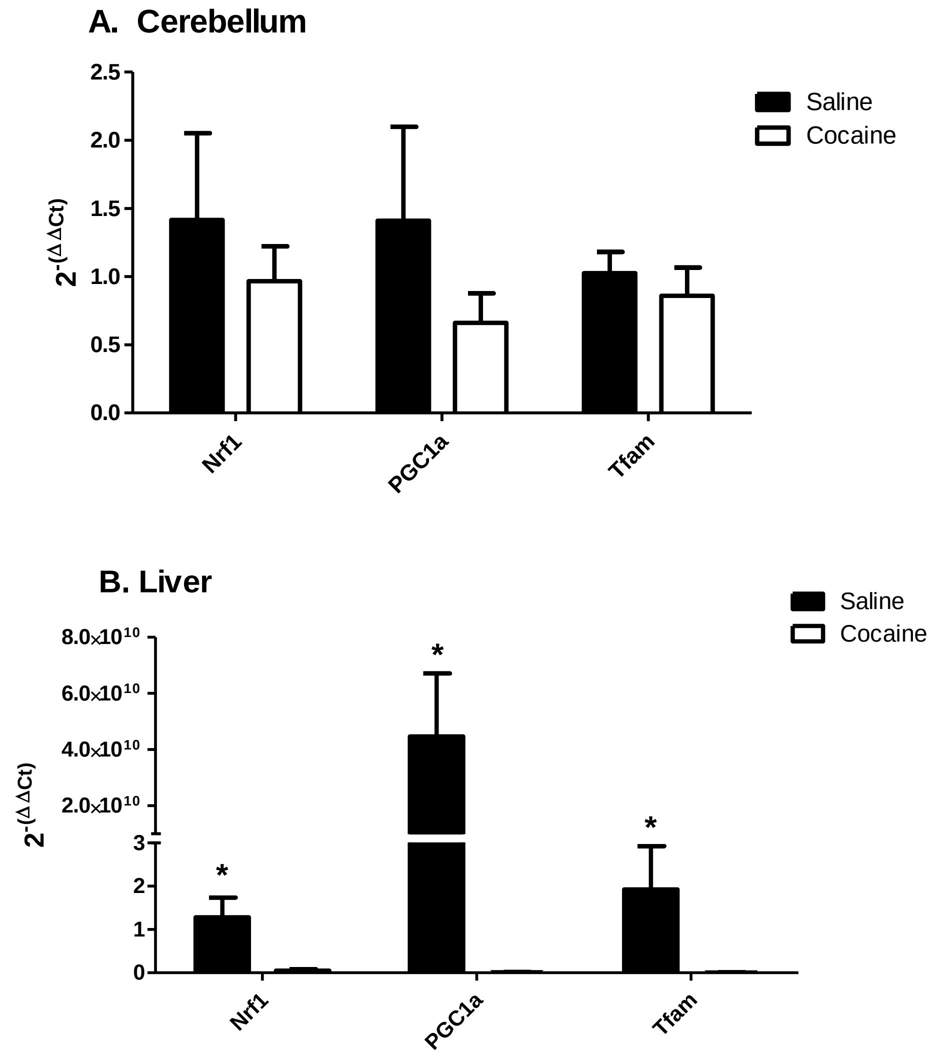

Supplement: Supplementary file 1 [file BRB3-9-e01451-s001.pdf]
